# Communication between cancer cell subtypes by exosomes contributes to nasopharyngeal carcinoma metastasis and poor prognosis

**DOI:** 10.1093/pcmedi/pbae018

**Published:** 2024-09-23

**Authors:** Hao-Jun Xie, Ming-Jie Jiang, Ke Jiang, Lin-Quan Tang, Qiu-Yan Chen, An-Kui Yang, Hai-Qiang Mai

**Affiliations:** Department of Head and Neck, Sun Yat-sen University Cancer Center, Guangzhou 510060, China; State Key Laboratory of Oncology in South China, Guangdong Key Laboratory of Nasopharyngeal Carcinoma Diagnosis and Therapy, Guangdong Provincial Clinical Research Center for Cancer, Sun Yat-sen University Cancer Center, Guangzhou 510060, China; Department of Head and Neck, Sun Yat-sen University Cancer Center, Guangzhou 510060, China; State Key Laboratory of Oncology in South China, Guangdong Key Laboratory of Nasopharyngeal Carcinoma Diagnosis and Therapy, Guangdong Provincial Clinical Research Center for Cancer, Sun Yat-sen University Cancer Center, Guangzhou 510060, China; Department of Head and Neck, Sun Yat-sen University Cancer Center, Guangzhou 510060, China; State Key Laboratory of Oncology in South China, Guangdong Key Laboratory of Nasopharyngeal Carcinoma Diagnosis and Therapy, Guangdong Provincial Clinical Research Center for Cancer, Sun Yat-sen University Cancer Center, Guangzhou 510060, China; Departcment of Nasopharyngeal Carcinoma, Sun Yat-sen University Cancer Center, Guangzhou 510060, China; State Key Laboratory of Oncology in South China, Guangdong Key Laboratory of Nasopharyngeal Carcinoma Diagnosis and Therapy, Guangdong Provincial Clinical Research Center for Cancer, Sun Yat-sen University Cancer Center, Guangzhou 510060, China; Departcment of Nasopharyngeal Carcinoma, Sun Yat-sen University Cancer Center, Guangzhou 510060, China; State Key Laboratory of Oncology in South China, Guangdong Key Laboratory of Nasopharyngeal Carcinoma Diagnosis and Therapy, Guangdong Provincial Clinical Research Center for Cancer, Sun Yat-sen University Cancer Center, Guangzhou 510060, China; Department of Head and Neck, Sun Yat-sen University Cancer Center, Guangzhou 510060, China; State Key Laboratory of Oncology in South China, Guangdong Key Laboratory of Nasopharyngeal Carcinoma Diagnosis and Therapy, Guangdong Provincial Clinical Research Center for Cancer, Sun Yat-sen University Cancer Center, Guangzhou 510060, China; Departcment of Nasopharyngeal Carcinoma, Sun Yat-sen University Cancer Center, Guangzhou 510060, China; State Key Laboratory of Oncology in South China, Guangdong Key Laboratory of Nasopharyngeal Carcinoma Diagnosis and Therapy, Guangdong Provincial Clinical Research Center for Cancer, Sun Yat-sen University Cancer Center, Guangzhou 510060, China

**Keywords:** cell heterogeneity, cell communication, exosomes, metastasis, nasopharyngeal carcinoma

## Abstract

**Background:**

Intratumor heterogeneity is common in cancers, with different cell subtypes supporting each other to become more malignant. Nasopharyngeal carcinoma (NPC), a highly metastatic cancer, shows significant heterogeneity among its cells. This study investigates how NPC cell subtypes with varying metastatic potentials influence each other through exosome-transmitted molecules.

**Methods:**

Exosomes were purified and characterized. MicroRNA expression was analyzed via sequencing and qRT-PCR. The effects of miR-30a-5p on migration, invasion, and metastasis were evaluated in vitro and in vivo. Its impact on desmoglein glycoprotein (DSG2) was assessed using dual-luciferase assays and Western blotting. Immunohistochemistry (IHC) and statistical models linked miR-30a-5p/DSG2 levels to patient prognosis.

**Results:**

Different NPC cell subtypes transmit metastatic potential via exosomes. High-metastatic cells enhance the migration, invasion, and metastasis of low-metastatic cells through exosome-transmitted miR-30a-5p. Plasma levels of exosomal miR-30a-5p are reliable indicators of NPC prognosis. miR-30a-5p may promote metastasis by targeting DSG2 and modulating Wnt signaling. Plasma exosomal miR-30a-5p inversely correlates with DSG2 levels, predicting patient outcomes.

**Conclusion:**

High-metastatic NPC cells can increase the metastatic potential of low-metastatic cells through exosome-transmitted miR-30a-5p, which is a valuable prognostic marker assessable via liquid biopsy.

## Graphical Abstract

**Graphical Abstract:**
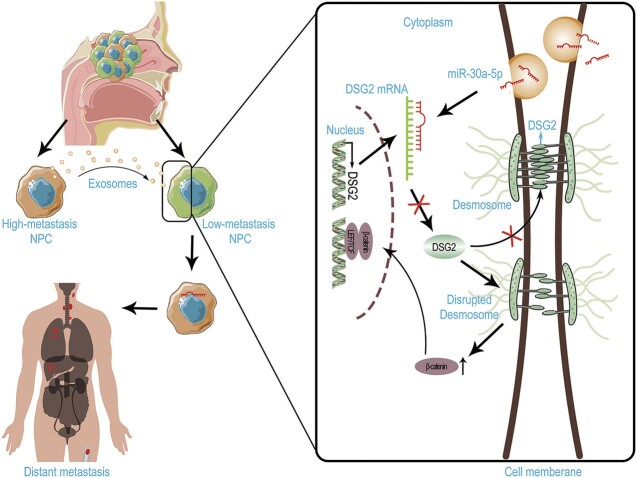

## Introduction

Nasopharyngeal carcinoma (NPC) is an epithelial carcinoma arising from the nasopharyngeal mucosal lining, with a distinct geographical distribution [1]. Despite significant progress in NPC treatment, the prognosis of NPC remains poor, particularly for patients with recurrent or metastatic disease [2]; and 10% of patients are diagnosed with distant metastasis at their initial diagnosis [1]. Advances in tumor biology research have highlighted the critical role of tumor heterogeneity in tumor initiation, progression, and therapy resistance [3, 4]. Single-cell sequencing has revealed significant heterogeneity within NPC, which may evolve during tumor progression [5, 6]. Nevertheless, the biological effects and underlying mechanisms of the heterogeneity remain to be uncovered.

Many researches have elucidated that heterogenous cancer cells can support each other, thereby enhancing malignancy. For instance, human lung adenocarcinomas displayed hierarchical features with two distinct subpopulations: one with high Wnt signalling activity and another forming a niche that provides the Wnt ligand; together, these subtypes enhance tumor progression [7]. Similarly, small-cell lung cancer generated its own microenvironment via activation of Notch signaling in a subset of tumor cells; although these cells were relatively slow growing, they contributed to chemoresistance and relapse after therapy [8]. These findings indicate that intratumor heterogeneous cells compensate for each other to achieve higher malignant biological behavior. However, whether the phenomenon exists in NPC remains unknown.

Heterogeneous cancer cells may communicate with each other via various routes, including direct cell–cell contact, soluble signaling molecules, extracellular vesicles (EVs) etc. Of note, EVs, comprising exosomes and microvesicles, have been found to play a vital role in mediating cell–cell communication in recent decades [9]. EV-mediated intercellular communication has been revealed to significantly contribute to promoting cancer initiation [10], progression [11], pre-metastatic niche formation [12], and therapy resistance [13, 14]. We have also demonstrated that therapy-induced dying-cell-derived exosomes promoted the recovery of damaged cells and tumor repopulation [15]. The role of EVs in NPC has also been underscored [16–18]. Nevertheless, the role of EVs in mediating heterogeneous cancer cell communication and their clinical significance in NPC remain unexplored.

We hypothesize that high metastatic NPC cells could influence the metastatic potential of low metastatic NPC cells, possibly via exosomes. In this study, we revealed that high metastatic NPC cell subclone-derived conditioned medium could enhance the migration and invasion capacity of low metastatic cells. Further experiments identified the vital role of exosomes in mediating this phenomenon. Exosomal miR-30a-5p significantly enhanced the metastatic capacity of the NPC cells. Of note, an elevated level of plasma exosomal miR-30a-5p in NPC patients was associated with poorer prognosis, including overall survival (OS), progression-free survival (PFS), distant metastasis-free survival (DMFS), and locoregional recurrence-free survival (LRFS). Mechanistically, high metastatic cell-derived exosomal miR-30a-5p significantly promoted tumor metastasis via suppressing desmoglein glycoprotein (DSG2) and further influencing Wnt/β-catenin signaling. Furthermore, DSG2 in the tumor tissue was statistically significantly correlated with the level of plasma exosomal miR-30a-5p and the prognosis of NPC patients. Our data revealed that high metastatic NPC cells conferred the aggressive potential to the low-capacity ones via exosomal miR-30a-5p, which significantly correlated with the prognosis of NPC patients and could be an ideal prognostic marker for these patients.

## Materials and methods

### Patient selection and grouping

A total of 130 NPC patients enrolled at our institution between 2011 and 2012 were selected as investigation candidates. All patients had been treated in the nasopharyngeal department of our hospital. Inclusion criteria were as follows: (i) >18 years old; (ii); World Health Organization case classification of type II or III; (iii) no history of distant metastasis; (iv) no history of anti-tumor therapy. Exclusion criteria were as follows: (i) lost to follow-up; (ii) previous or current history of other malignant tumors; (iii) incomplete case data; (iv) current pregnancy or lactation. A total of 119 patients were included in the study after excluding patients who did not meet the criteria. Plasma samples and pathological tissue sections of these patients were collected. The study was approved by the Ethical Committee of Sun Yat-sen University Cancer Center and informed consent was obtained from all patients.

Plasma exosomal RNA was extracted for qRT-PCR and the patients were divided into two groups by the median value of plasma exosomal miR-30a-5p expression level.

### Cell lines and culture

The NPC cell line CNE-2, along with the high metastatic subtype S18 and low metastatic subtype S26, were gifts from Professor Qian [19]. These cells are cultured with RPMI 1640 supplemented with 5% fetal bovine serum (FBS). Human embryonic kidney HEK-293T cells were purchased from ATCC and maintained in our laboratory. HEK-293T cells were cultured in Dulbecco's modified eagle medium (DMEM) supplemented with 10% FBS. All the cells were incubated in a humidified incubator containing 5% CO_2_ at 37°C and all the cell lines were routinely tested to ensure they were mycoplasma-free.

### *In vivo* metastasis assay

The lung metastasis model was established via tail-vein injection of cancer cells. In detail, cells that overexpressed miR-30a-5p or control sequence, and cells that were pre-treated with exosomes derived from miR-30a-5p overexpression or control cells, were used. The cells were digested and diluted to a concentration of 1×10^7^/ml, and 200 μl of the cell suspensions was injected into each mouse via the tail vein. After 40 days, whole blood was collected from the orbit sinus and the mice were sacrificed to collect the lung tissue. Exosomes were extracted from the plasma for further analysis. The weight and nodule numbers of the lungs were recorded, and the lungs were then paraffin embedded and further hematoxylin-eosin (H&E) staining was performed. The animal experimental protocol was approved by the Animal Ethics Committee of Sun Yat-sen University Cancer Center.

### Exosome extraction

Medium containing exosome-depleted FBS was used for cell culture. After 48 h, the medium was collected and differential centrifugation was used to isolate exosomes. The protocol for differential centrifugation involved the following steps: centrifugation at 300 g at 4 °C for 10 min, 2000 g at 4°C for 20 min, and 110 000 g at 4°C for 2 h. The pallet was collected and resuspended in phosphate-buffered saline (PBS), following by an additional centrifugation at 110 000 g at 4°C for 2 h. The exosome pallet was collected for further experiment or analysis.

Plasma exosomes were extracted using a Total Exosome Isolation Kit (from plasma) (Thermal Fisher Scientific) following the manufacturer's instructions. Specifically, 200 μl of plasma was first centrifuged at 2000 g at 4°C for 20 min, followed by centrifugation at 10 000 g at 4°C for 20 min. The supernatant was then diluted with 100 μl of PBS, and 5 μl of proteinase K was added for digestion at 37°C for 10 min. Then, 60 μl of exosome precipitation reagent (from plasma) was added and incubated at 4°C for 30 min. After centrifugation at 10 000 g at 4°C for 5 min, the exosome pallet was resuspended with PBS and further purified by a 100 kDa ultrafiltration device.

### Analysis of exosomes

For transmission electron microscopy (TEM) analysis, exosomes were placed on copper grids and stained with phosphotungstic acid. After the exosomes dried, images were taken at an operating voltage of 100 kV. For nanoparticle tracking analysis (NTA), exosomes were resuspended in PBS and detected by NanoSight NS300 (Malvern Panalytical). For the detection of exosomal proteins by immunoblotting, exosomes were treated similarly to cells for the extraction of proteins.

When testing the ingestion of exosomes by recipient cells, exosomes were diluted with Diluent C and then stained with PKH67 for 4 min. After termination of the staining with PBS containing 0.5% BSA, the exosomes were washed with PBS four times. Exosomes (20 µg/ml) were added to the recipient cells and incubated at 37°C for 8 h. The cells were then fixed with 4% paraformaldehyde, stained with 4',6-diamidino-2-phenylindole (DAPI), and imaged with a laser confocal scanning microscope.

### Cell migration and invasion assay

Corning Matrigel Invasion Chamber (Corning 354 480) inserts were used for invasion assays, while Transwell® inserts (8 μm PET membrane, Corning 3464) without Matrigel matrix coating were used for migration assays.

Cells were counted and diluted to a density of (2–5) × 10^5^/ml with serum-free medium. A total of 200 μl of the cell suspension was seeded into the upper chamber of each insert. Culture medium (800 μl) with 10% FBS was added to the lower chambers. The cells were cultured for 24 h and then fixed by 4% paraformaldehyde. Next, the cells were stained with crystal violet for 10 min. Cells on the inside of the Transwell inserts were gently removed using cotton swabs, and cells on the lower surface of the membrane were observed and imaged under a microscope.

### qPCR detection of micro RNA (miRNA) expression

RNA was extracted from cell or exosome samples by TRIzol reagent (Invitrogen) following the manufacturer's instructions. The RNA was reverse transcribed with the specific reverse transcription primer and the PrimeScript™ RT reagent kit (Takara) following the manufacturer's instructions. The primer sequence used for reverse transcription was as follows: 5′-CTCAACTGGTGTCGTGGAGTCGGCAATTCAGTTGAGCTTCCA-3′ for hsa-miR-30a-5p, 5′-CTCAACTGGTGTCGTGGAGTCGGCAATTCAGTTGAGCAAGCT-3′ for cel-miR-39a-3p, 5′-CTCAACTGGTGTCGTGGAGTCGGCAATTCAGTTGAGAAAAATAT-3′ for RUN6-1 (U6 small nuclear RNA). qPCR was performed with the TB Green Premix Ex Taq Kit (Takara) following the manufacturer's instructions. Primers used for the qPCR assay were as follows: has-miR-30a-5p forward: 5′-ACTCAGCTGGTGTAAACATCCTCGAC-3′; has-miR-30a-5p reverse: 5′-TGGTGTCGTGGAGTCG-3′; cel-miR-39a-3p forward: 5′-ACACTCCAGCTGGGTCACCGGGTGTAAATC-3′; cel-miR-39a-3p reverse: 5′- TGGTGTCGTGGAGTCG-3′; RUN6-1 forward: 5′-CAAGGATGACACGCAAA-3′; RUN6-1 reverse: 5′-TCAACTGGTGTCGTGG-3′. The relative gene expression was calculated by the 2^−ΔΔCt^ algorithm.

### Dual luciferase reporter assay

The Dual-Luciferase^®^ Reporter (DLR™) Assay System (Promega) was applied to verify the target sites of miR-30a-5p in DSG2. Briefly, CNE-2 cells were co-transfected with pmirGLO plasmids containing either the miR-30a-5p target site or its mutant form, along with miR-30a-5p mimic or control, using Lipofectamine 3000 according to the manufacturer's protocol. Cells were collected 48 h later and analyzed for dual luciferase activities following the manufacturer's protocol. Luciferase activities were calculated by the ratio of firefly to renilla luminescence.

### Immunohistochemistry staining

Tissue sections were subjected to dewaxing with xylene and a serial concentration of ethanol (95%, 90%, 80%, 70%, 60%, 0% for 5 min each) and then blocked with 3% H_2_O_2_ for 20 min. Antigen retrieval was achieved in boiling citrate antigen retrieval solution (pH = 6.0) for 2.5 min under pressure. After washing with PBS, the sections were incubated overnight at 4°C with the primary antibody, followed by incubation with the secondary antibody at 37°C for 20 min. Immunohistochemistry was completed by 3,3'-diaminobenzidine (DAB) staining followed by hematoxylin staining, according to the manufacturer's protocol. Images of the sections were taken and scored. An initial score 0 means negative for staining, score 1 means weak positive, score 2 means medium positive, score 3 means strong positive. The percentage of positive cells was also recorded. The final score was calculated by multiplying the initial score by the positive rate.

### miRNA sequencing

High-throughput sequencing of exosomal miRNAs was performed by RiboBio (Guangzhou, China). Total RNAs of exosomes derived from S18, S26, and CNE-2 cells were used to prepare the miRNA sequencing libraries and ∼150 bp PCR amplicons (corresponding to ∼22 nt miRNAs) were selected. The libraries were then sequenced with Illumina HiSeq sequencer (Illumina) following standard protocols.

### Statistics

All data were analyzed with SPSS 25.0 and GraphPad Prism 7. Normally distributed data are presented as mean ± standard deviation (SD). Differences between means were assessed using the unpaired student's t test. Survival analysis was performed by the Kaplan–Meier method. Multivariable survival analyses were conducted using the Cox proportional hazards model. *P *< 0.05 was considered statistically significant. Pearson's correlation coefficient (*r* value) was calculated assuming a linear relationship between variables.

## Results

### High metastatic NPC cell-derived exosomes confer migration and invasion ability on low metastatic cells

**Figure 1.**
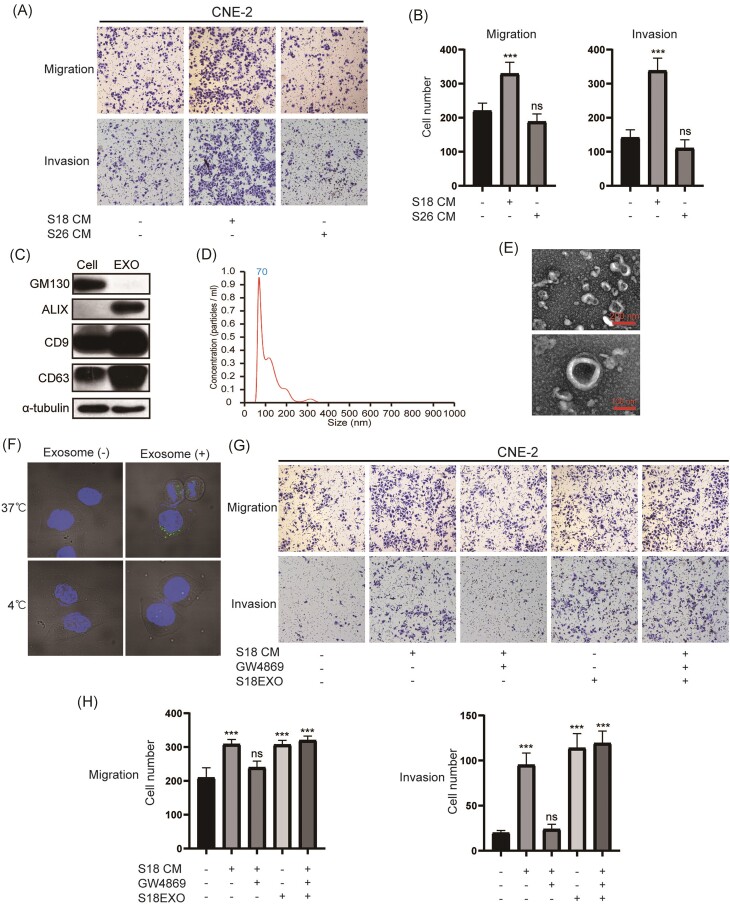
High metastatic NPC cell-derived exosomes confer migration and invasion ability on low metastatic cells. Images (**A**) and statistical diagrams (**B**) showing the migration and invasion ability of CNE-2 cells treated with CM from different subtypes of cells. (**C**) Western blot identification of exosome markers including ALIX, CD9, and CD63; the cell marker GM130 was used as a negative control. (**D**) Diagram showing the distribution of diameter of the exosomes detected by Nanosight NS300. (**E**) TEM images detecting phosphotungstic acid-stained exosomes. (**F**) Fluorescent images of the CNE-2 cells treated with exosomes stained with PKH67. Images (**G**) and statistical diagrams (**H**) showing the migration and invasion ability of CNE-2 cells treated with the indicated components. ns, *p*>0.05; ***, *p*<0.001.

To investigate whether the heterogeneity among NPC cells influences their metastatic capacity, we employed two subclones of NPC cells derived from CNE-2: subclone 18 (S18, with high metastatic potential) and S26 (with low metastatic potential) [19]. Using conditioned medium (CM) from these subclones to treat CNE2 cells, we found that CM from high metastatic S18 dramatically promoted the migration and invasion capacity of treated cells (Fig. 1A and B). As numerous studies revealed the vital role of exosomes in mediating cellular communication, we isolated exosomes from the CM of these cells using gradient ultracentrifugation. The isolated exosomes were characterized using TEM, NTA, and western blotting as previously described [15]. The results supported that the characteristics of isolated exosomes matched the definition of exosomes, validating the reliability of our isolation methods (Fig. 1C–E). Further experiments confirmed the uptake of exosomes by the recipient cells (Fig. 1F). Similar to the effect of S18 CM, S18 cell-derived exosomes also enhanced the migration and invasion of NPC cells. However, CM derived from cells treated with GW4869, a compound that inhibits exosome secretion [20], showed impaired capacity to promote cell migration and invasion (Fig. 1G and H). Furthermore, addition of exosomes to GW4869-treated CM restored the capacity to promote cell migration and invasion (Fig. 1G and H). Taken together, these results illustrate that high metastatic S18 cell-derived exosomes can confer metastatic ability on lower metastatic cells.

### miR-30a-5p is significantly upregulated in S18-derived exosomes and exosome-treated low metastatic cells

**Figure 2.**
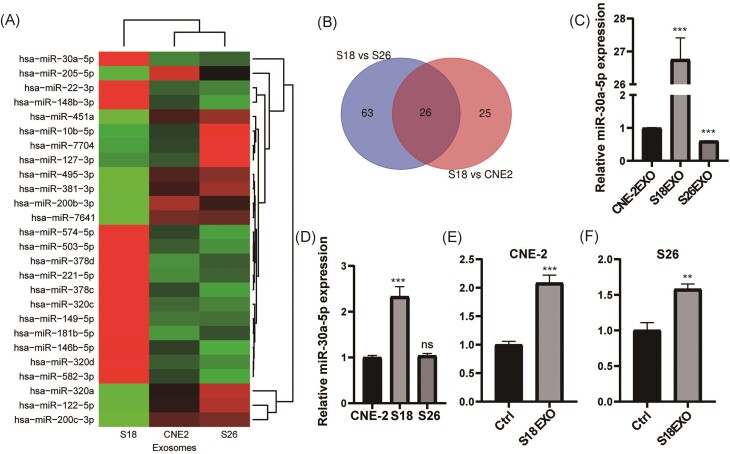
miR-30a-5p is significantly upregulated in S18-derived exosomes and the exosome-treated low metastatic cells. (**A**) Heatmap of the differentiation expressed miRNAs in exosomes derived from different subtypes of cells. (**B**) Venn diagram showing the overlapped miRNAs that are elevated in S26 cells compared with both S18 cells and CNE-2 cells. (**C**) qPCR detection of miR-30a-5p expression in exosomes derived from different subtypes of cells. (**D**) Relative expression of miR-30a-5p in different subtypes of cells by qPCR. qPCR detection of miR-30a-5p expression in CNE-2 (**E**) and S26 (**F**) cells treated with exosomes derived from S18 cells. ns, *p*>0.05; **, *p*<0.01; ***, *p*<0.001.

To investigate the underlying mechanisms by which S18-derived exosomes potentiate metastasis, we analyzed the contents of these exosomes by high-throughput sequencing. Due to the vital role of miRNAs in mediating the effects of exosomes [21], we focused on analyzing miRNAs. miRNA sequencing revealed that 26 miRNAs were upregulated in S18 cell-derived exosomes compared with both S26 cells and the parental CNE2 cells (Fig. 2A and B, supplementary Table 1, see supplementary online material). Among these, miR-30a-5p was one of the highest upregulated miRNAs in S18-derived exosomes, a finding that was confirmed by qPCR analysis (Fig. 2C). In addition, miR-30a-5p was also upregulated in S18 cells compared with both S26 and the parental CNE-2 cells (Fig. 2D). Of note, the expression of miR-30a in S26 and CNE-2 cells was significantly upregulated following treatment with exosomes derived from S18 cells (Fig. 2E and F). These results indicate that the upregulated miR-30a-5p in S18 cells can be transmitted to S26 and CNE-2 cells via exosomes. Thus, we selected miR-30a-5p for further investigation.

### Lower plasma exosomal miR-30a-5p level predicts better prognosis of NPC patients

Given that miR-30a-5p was selected by investigation from the *in vitro* cell models, we investigated its clinical significance in NPC patients. Plasma exosomal miRNA was extracted from selected patients as described in the Materials and methods section, and the relationship between exosomal miR-30a-5p and clinical features of NPC patients was analyzed. A total of 119 NPC patients was divided into two group based on the expression level of exosomal miR-30a-5p. The basic information on NPC patients is presented in supplementary Table 2 (see supplementary online material), which shows no significant differences between the high and low exosomal miR-30a-5p groups. After a median follow-up of 84.6 months (range: 3.9–108.7 months), 17 patients encountered local regional relapse, 32 with metastasis, 46 with disease progression, and 23 deaths. We selected OS as the primary investigation endpoint, and PFS, DMFS, and LRFS as the secondary endpoint.

**Figure 3.**
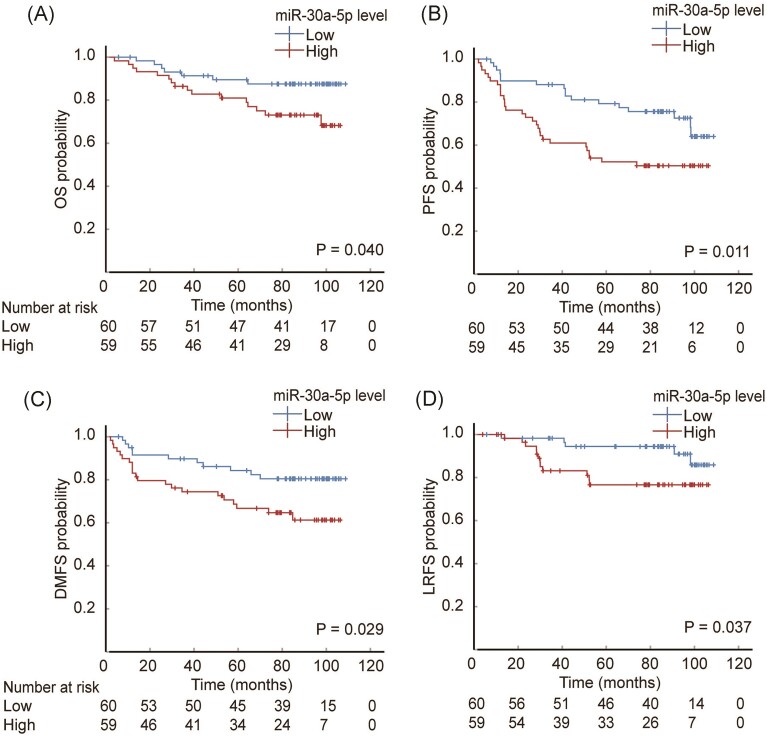
Lower plasma exosomal miR-30a-5p level predicts better prognosis of NPC patients. Kaplan–Meier survival curves showing the association between the differential expression of plasma exosomal miR-30a-5p and (**A**) OS, (**B**) PFS, (**C**) DMFS, and (**D**) LRFS.

The results show that lower plasma exosomal miR-30a-5p was associated with better 5-year OS of NPC patients; the lower plasma exosomal miR-30a-5p group has a 5-year survival rate of 89.5% [95% confidence interval (CI) 81.5%–97.5%], and the higher group of 81.0% (95% CI 70.9%–91.1%), with *P* value of 0.04 (Fig. 3A). Moreover, lower plasma exosomal miR-30a-5p level correlated with better PFS [79.3% (95% CI 68.8%–89.8%) versus 52.2% (95% CI 39.5%–64.9%), *P *= 0.011] (Fig. 3B), better DMFS [84.3% (95% CI 74.8%–93.7%) versus 66.7% (95% CI 54.4%–70.0%), *P *= 0.029] (Fig. 3C), and better LRFS [94.4% (95% CI 88.3%-100.0%) versus 76.6% (95% CI 65.0%–88.3%), *P *= 0.037] (Fig. 3D). In multivariate models adjusted for relevant confounders, higher plasma exosomal miR-30a-5p level remained an independent risk factor for poorer PFS [hazard ratio (HR) = 1.976, 95% CI 1.061–3.680, *P *= 0.032] and DMFS (HR = 2.245, 95% CI 1.034–4.872, *P *= 0.041), but not for poorer OS (HR = 2.333, 95% CI 0.928–5.865, *P *= 0.072) or LRFS (HR = 2.569, 95% CI 0.874–7.555, *P *= 0.086) with a *P*-value nearly reaching statistical significance (supplementary Table 3, see supplementary online material). These results strongly support that plasma exosomal miR-30a-5p is a reliable indicator of the prognosis of NPC patients, and suggest that exosomal miR-30a-5p may be vital in regulating tumor biology, such as transmitting the metastatic potential of heterogeneous NPC cells.

### Exosomal miR-30a-5p enhanced the migration and invasion ability of NPC cells

**Figure 4.**
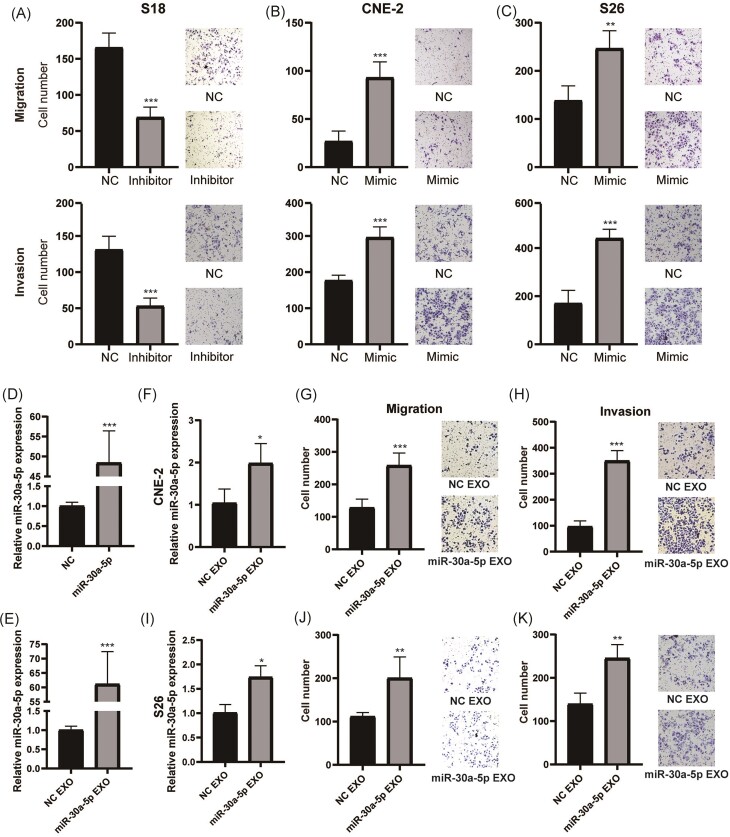
Exosomal miR-30a-5p enhanced migration and invasion ability of NPC cells. (**A**) The ability of cell migration (top) and invasion (bottom) after transfection of miR-30a-5p inhibitor in S18 cells. Representative images are shown on the right. The ability of cell migration (top) and invasion (bottom) after transfection of miR-30a-5p mimic in CNE-2 (**B**) and S26 (**C**) cells. Representative images are shown on the right. (**D**) Relative expression of miR-30a-5p in miR-30a-5p overexpression or control cells. (**E**) Relative expression of miR-30a-5p in exosomes derived from miR-30a-5p overexpression and control cells. (**F**–**H**) After treatment with exosomes from miR-30a overexpression and control cells, the expression of miR-30a-5p (F), migration (G), and invasion (H) ability of CNE-2 cells. Representative images are shown on the right. (**I**–**K**) After treatment with exosomes from miR-30a overexpression and control cells, the expression of miR-30a-5p (I), migration (J), and invasion (K) ability of S26 cells. Representative images are shown on the right. *, *p*<0.05; **, *p*<0.01; ***, *p*<0.001.

We further analyzed the biological role of miR-30a-5p in regulating the metastatic potential of NPC cells. Transfection of miR-30a-5p inhibitor in S18 cells significantly inhibited cell migration and invasion (Fig. 4A; Supplementary Fig. 1A, see supplementary online material). Conversely, transfection of miR-30a-5p mimic enhanced the migration and invasion capacities in S26 and CNE-2 cells (Fig. 4B and C; supplementary Fig. 1B and C), similar to the effect of S18-derived exosomes.

To further mimic the role of S18 cell-derived exosomal miR-30a-5p, we constructed an miR-30a-5p stable overexpression cell line in CNE-2 cells (Fig. 4D). The exosomes derived from the miR-30a-5p overexpression cell line also showed enhanced expression of miR-30a-5p (Fig. 4E), similar to exosomes derived from S18 cells. Furthermore, CNE-2 and S26 cells that were treated with exosomes derived from the miR-30a-5p overexpression cell line also showed upregulation of miR-30a-5p (Fig. 4F and I). Consequently, CNE-2 and S26 cells treated with these exosomes exhibited significantly elevated migration and invasion capacities, respectively (Fig. 4G, H, J, and K). Taken together, these data demonstrate that exosomal miR-30a-5p is responsible for the enhanced migration and invasion capacities transmitted from high metastatic to low metastatic cells.

### Exosomal miR-30a-5p enhanced metastasis of cells *in vivo*

**Figure 5.**
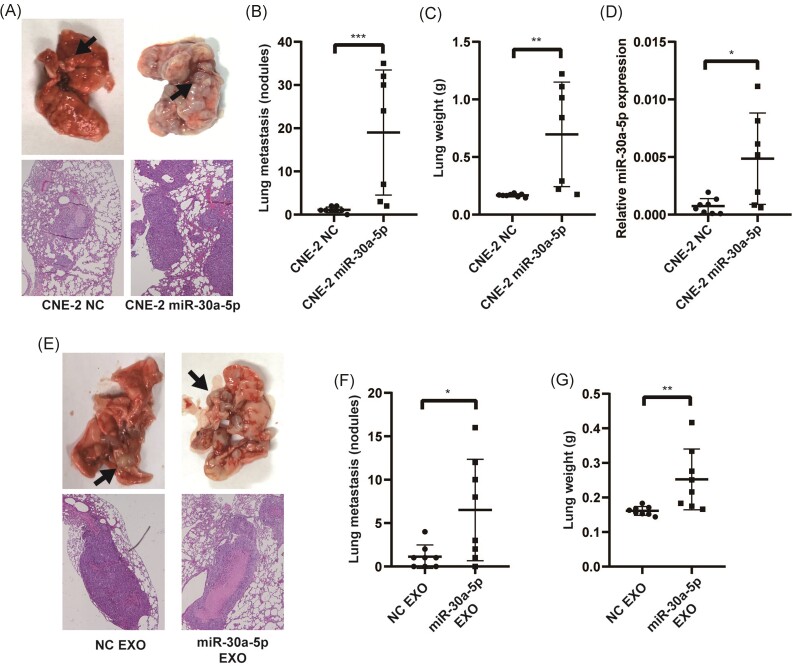
Exosomal miR-30a-5p enhanced metastasis of cells *in vivo*. Representative images (**A**) and statistics (**B**) of lung metastasis nodules in mice intravenously injected with miR-30a-5p overexpression or control cells. (**C**) Lung weight of mice intravenously injected with miR-30a-5p overexpression or control cells. (**D**) Relative expression of miR-30a-5p in the metastatic nodules of different experimental groups. Representative images (**E**) and statistics (**F**) of lung metastasis nodules in mice intravenously injected with CNE-2 cells pre-treated with exosomes derived from miR-30a-5p overexpression or control cells. (**G**) Lung weight of mice intravenously injected with CNE-2 cells pre-treated with exosomes derived from miR-30a-5p overexpression or control cells. *, *p*<0.05; **, *p*<0.01; ***, *p*<0.001.

To further explore the effect of miR-30a-5p on potentiating NPC cell metastasis, we injected CNE-2 miR-30a-5p overexpression cells and control cells via the tail vein in Balb/c nude mice. After ∼6 weeks, the mice were euthanized and their lungs were analyzed. Lungs from mice injected with miR-30a-5p overexpression cells showed significantly more metastatic nodules and H&E staining confirmed the significant infiltration of metastatic tumor cells nodules (Fig. 5A and B). Moreover, the lungs from mice injected with miR-30a-5p overexpression cells were significantly heavier than those from the control group (Fig. 5C). In addition, qPCR confirmed the overexpression of miR-30a-5p in the metastatic nodules of the miR-30a-5p overexpression group (Fig. 5D). Taken together, these results indicate that elevated miR-30a-5p expression in NPC cells enhanced tumor metastasis.

Furthermore, we directly isolated exosomes from the miR-30a-5p overexpression and the control cells and used them to treat CNE2 cells. After that, the treated cells were injected into nude mice via the tail vein. The results showed that miR-30a-5p overexpression exosome-treated CNE-2 cells exhibited enhanced metastatic potential (Fig. 5E). More metastatic nodules were observed and the lungs were heavier in the miR-30a-5p overexpression exosome-treated group (Fig. 5F and G). These results strongly support that exosomal miR-30a-5p enhanced the metastatic capacity of the recipient cells.

### miR-30a-5p targets DSG2 to enhance the metastatic capacity of NPC cells

**Figure 6.**
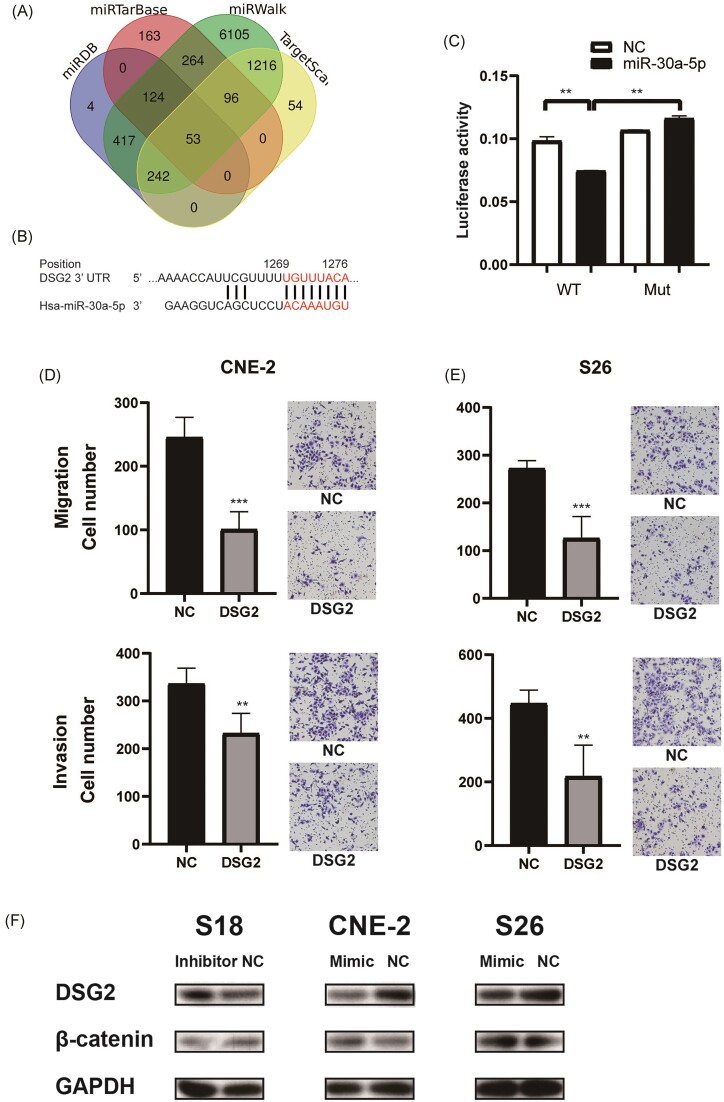
miR-30a-5p targets DSG2 to enhance the metastatic capacity of NPC cells. (**A**) Venn diagram showing the overlap of predicted miR-30a-5p target genes by different algorithms. (**B**) Schematic diagram of the potential targeting site of miR-30a-5p in the 3′UTR of DSG2. (**C**) Results of the dual luciferase assay reporter system, which show that miR-30a-5p inhibited the translation of DSG2, and the repression effect is abrogated when the predicted binding sequence is mutated. Transwell assay showing the migration (top) and invasion (bottom) of the CNE2 (**D**) and S26 (**E**) cells transfected with DSG2 overexpression or control plasmid. Representative images are shown on the right. (**F**) Western blot detecting the expression of DSG2 and β-catenin in S18 cells transfected with miR-30a-5p inhibitor, or in CNE2 and S26 cells transfected with miR-30a-5p mimic. **, *p*<0.01; ***, *p*<0.001.

miRNAs exert their regulatory function mainly by targeting the downstream mRNAs via the 3′-untranslated region (3′-UTR). We used several miRNA target-predicting algorithms, including miRDB, miRTarBase, miRWalk, and TargetScan. Venn diagram analysis revealed that 53 targets were predicted to be the direct target of miR-30a-5p by all four algorithms (Fig. 6A). Among these potential targets, we identified the desmoglein glycoprotein DSG2, the main component of desmosome, which plays a vital role in cell connection and suppressing tumor metastasis [22, 23], as a target of miR-30a-5p. A dual-luciferase reporter assay demonstrated that miR-30a-5p inhibited DSG2 via binding to the predicted binding site (Fig. 6B and C).

To further test the function of DSG2 downstream of miR-30a-5p, we transfected DSG2 overexpression plasmids into NPC cells including high metastatic S26 cells and the parental CNE-2 cells. In contrast to miR-30a-5p overexpression, DSG2 overexpression significantly inhibited the migration and invasion ability of NPC cells (Fig. 6D and E).

As an important desmosome component, DSG2 inhibition by exosomal miR-30a-5p may disrupt the formation of desmosomes between cells. This may lead to the translocation of desmosomes to the cytosome/nucleus and alter cell signaling, such as activating Wnt signaling in head and neck squamous cell carcinoma [24]. We then transfected miR-30a-5p mimic and inhibitor into NPC cells and tested their influence on the expression of DSG2 and Wnt signaling. The results showed that miR-30a-5p inhibitor elevated the expression of DSG2 and downregulated β-catenin expression, while miR-30a-5p mimic transfection inhibited the expression of DSG2 and elevated β-catenin (Fig. 6F). These results indicated that exosomal miR-30a-5p could inhibit DSG2 expression, which further regulated Wnt signaling to promote cell metastasis.

### DSG2 is inversely correlated with miR-30a-5p expression and predicts better prognosis

**Figure 7.**
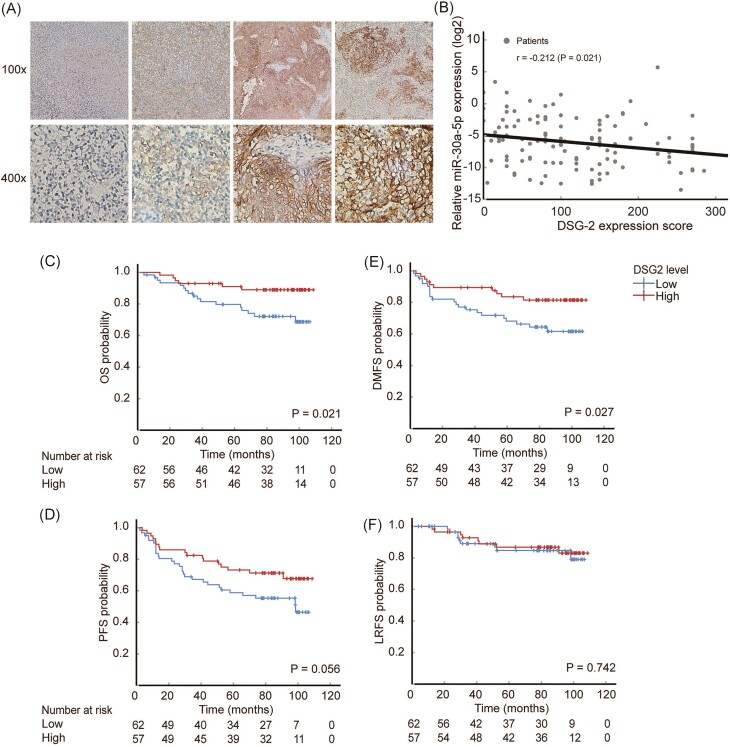
DSG2 is inversely correlated with the expression of miR-30a-5p and predicts better prognosis. (**A**) Representative images of immunohistochemistry staining of NPC tissues. The upper row shows images with amplification of 100x, while the lower row shows the 400x. The images displayed from left to right show negative to strong positive staining of DSG2 in NPC tissues. (**B**) Correlation of plasma exosomal miR-30a-5p level and tissue DSG2 expression level. (**C**–**F**) Kaplan–Meier survival curves showing the associations between the differential expression of DSG2 and (C) OS, (D) DMFS, (E) PFS, and (F) LRFS.

We further investigated the relationship between DSG2 and miR-30a-5p expression in clinical settings. The expression of DSG2 in patient samples was detected by immunohistochemistry (Fig. 7A). Surprisingly, the expression of DSG2 was significantly inversely correlated with the level of plasma exosomal miR-30a-5p (Fig. 7B). Moreover, lower DSG2 expression, which is associated with higher levels of plasma exosomal miR-30a-5p, was significantly associated with worse OS for the patients [79.7% (95% CI 69.5%–90.0%) in the lower DSG2 group versus 91.0% (95% CI 83.5%–98.5%) in the higher DSG2 group, *P *= 0.021] (Fig. 7C). Additionally, lower DSG2 expression was associated with worse 5-year DMFS [68.1% (95% CI 56.2%–80.0%) versus 83.5% (95% CI 73.6%–93.4%), *P *= 0.027] (Fig. 7D).

Although the differences in 5-year PFS was not statistically significant, there was a trend toward correlation [58.8% (95% CI 46.4%–71.2%) in the lower DSG2 group versus 73.2% (95% CI 61.5%–84.8%) in the higher DSG2 group, *P *= 0.056] (Fig. 7E). In addition, the 5-year LRFS was similar between the two groups [84.7% (95% CI 74.9%–94.5%) versus 86.8% (95% CI 77.7%–95.9%), *P *= 0.742] (Fig. 7F). These results revealed DSG2 as a complementary marker for predicting the prognosis of NPC patients and strongly support the crucial role of miR-30a-5p/DSG2 signaling in regulating NPC cell metastasis.

## Discussion

**Figure 8.**
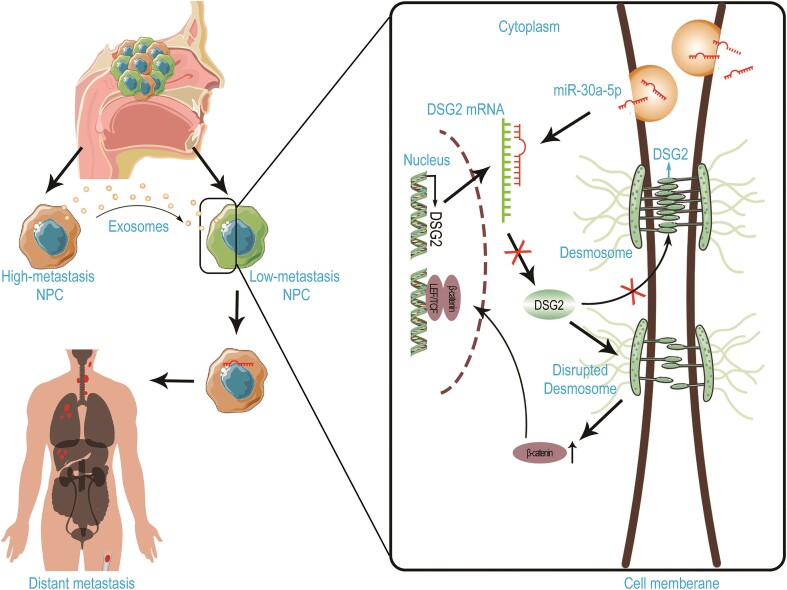
Schematic diagram depicting how high metastatic NPC cell-derived exosomal miR-30a-5p promotes the metastatic potential of low metastatic cells via suppressing DSG2 and enhancing Wnt signaling.

In this manuscript, we revealed that high metastatic NPC cells could enhance the metastatic capacity of low metastatic cells via exosome-based cell communication. High metastatic cell-derived exosomes contain large amounts of miR-30a-5p, which significantly promoted NPC cell metastasis by targeting DSG2 and regulating Wnt signaling (Fig. 8). Of note, a higher level of plasma exosomal miR-30a-5p was significantly associated with poor survival of the patients, and was negatively correlated with the expression of DSG2 in tumor tissues, which also correlated with patient survival. To the best of our knowledge, this is the first study to demonstrate that communication between heterogeneous NPC cells via exosomes promotes cancer metastasis and that this mechanism could be a prognostic marker for NPC patients. Translating these findings into therapeutic interventions presents promising avenues for improving patient outcomes in NPC; targeting miR-30a-5p by antisense oligonucleotides or miRNA sponges, modulating exosome production and release, and inhibiting wWt signaling are all worth further investigation.

Tumor cell heterogeneity is universal among cancers, and single cell RNA-sequencing has even revealed that every cancer cell could be different within a single patient [25]. Previous investigations have revealed that communication between different subtypes of cancer cells collectively promoted cancer progression [3, 4]. Moreover, loss of certain abilities in a cell may be compensated for by an increase in others. For example, cells that are slow-growing may be more therapy resistant, a phenomenon that has been exploited to develop adaptive therapy [26, 27]. In this scenario, the low metastatic NPC cells may be more therapy resistant or more fast growing when their metastatic potential is enhanced by high metastatic cells, and the cancer may become even more malignant.

As an important communicating cargo, exosomes have been revealed to mediate the communications between different subtypes and promote cancer cell progression. *In vivo* imaging revealed that EVs released from malignant tumor cells are taken up by less malignant tumor cells, and the EV-delieved mRNAs promoted migration and metastasis of these cells [28]. Moreover, therapy-resistant renal cancer cells could transmit their therapy resistant potential to sensitive cells via exosome-delivered long non-coding RNA (lncRNA) [29]. In this article, we further revealed that high metastatic cancer cells could transmit their metastatic potential to low metastatic NPC cells via delivering exosomes, which significantly promoted cancer cell metastasis.

miRNAs are short non-coding RNAs that modulate the expression of genes to exert their biological function and are a vital component of exosomes. Exosomally-delivered miRNAs can modulate various aspects of tumor biology and are a vital messenger within the tumor microenvironment [30]. Our colleagues have also revealed that miR-30a-5p is associated with the malignant progression of NPC [31], although the underlying mechanisms remain undiscovered. In this study, we revealed that high metastatic NPC cell-derived exosomal miR-30a-5p could promote the migration, invasion, and metastatic potential of low metastatic cancer cells, which could be a significant factor in cancer progression. The mechanism by which miRNAs exert their biological functions involves binding to the 3′UTR of mRNAs and inhibiting translation, thus inhibiting gene expression [32]. Here, we illustrated that miR-30a-5p inhibited the expression of DSG2 via binding to the seed sequence in the 3′UTR. As an important component of the desmosome, DSG2 inhibition has been found to elevate epithelial–mesenchymal transition gene expression, allowing cells to detach from the primary tumor and undergo intravasation [22]. DSG2 inhibition was also revealed to promote cell invasion and migration, which is associated with Wnt signaling [33]. Furthermore, DSG2 has been identified as a prognostic marker in several cancers [34]. These previous investigations support our findings that miR-30a-5p could promote cancer metastasis via targeting DSG2 and regulating Wnt signaling.

Liquid biopsy has gained significant attention recently due to its convenience, minimal invasiveness, and accuracy in diagnosis, especially in cancer diagnosis and prognosis prediction [35]. Among the tested components, exosomes are promising for liquid biopsy [36]. However, most identified liquid biopsy markers are based on screening for a previously identified molecule whose biological functions and mechanisms are not fully understood. In this study, based on findings from bench work, we revealed that the metastatic promoting exosomal miR-30a-5p could be an important prognosis predicting marker for NPC patients, which was associated with the tissue expression of DSG2. The synergy between bench and bedside work collectively illustrates that exosomal miR-30a-5p is a strong candidate for use as a liquid biopsy marker. This approach underscores the potential of integrating laboratory findings with clinical applications to improve cancer diagnosis and prognosis.

While our study provides significant insights into the role of exosomal miR-30a-5p in NPC metastasis and prognosis, it is important to recognize its limitations. One of the limitations of our study is the sample size. We included 119 NPC patients in our analysis. While this sample size provided sufficient power to detect significant associations between plasma exosomal miR-30a-5p levels and patient prognosis, it may limit the generalizability of our findings to the broader NPC patient population. Larger, multi-center studies will be necessary to validate our results. Moreover, although we identified miR-30a-5p as a key mediator of metastasis via targeting DSG2 and modulating Wnt signaling, the exact molecular mechanisms and interactions remain to be fully elucidated. Additional studies are needed to dissect these pathways further and to explore other potential targets of miR-30a-5p that may contribute to NPC progression.

In summary, we revealed that a high metastatic subtype of NPC cells can transmit their metastatic potential to low metastatic cells via secreting exosomal miR-30a-5p. This miRNA targets DSG2 to regulate Wnt signaling and finally promote metastasis. Plasma exosomal miR-30a-5p is a promising marker for predicting the prognosis of NPC patients. This study uncovers the intercellular communication between different subtypes of NPC cells that promotes cancer progression and provides a potential biomarker for predicting metastatic risk.

## Supplementary Material

pbae018_Supplemental_Files

## Data Availability

The miRNA sequencing data have been submitted to the NCBI Sequence Read Archive (SRA) under the accession number PRJNA1051654.
