# Supplementary material for: Communication between cancer cell subtypes by exosomes contributes to nasopharyngeal carcinoma metastasis and poor prognosis
Source: Precis Clin Med. 2024 Sep 23;7(3):pbae018. doi: 10.1093/pcmedi/pbae018 (PMC11427951; doi:10.1093/pcmedi/pbae018)
Supplement: pbae018_Supplemental_Files [file pbae018_supplemental_files.zip › Supplementary Figure1.docx]

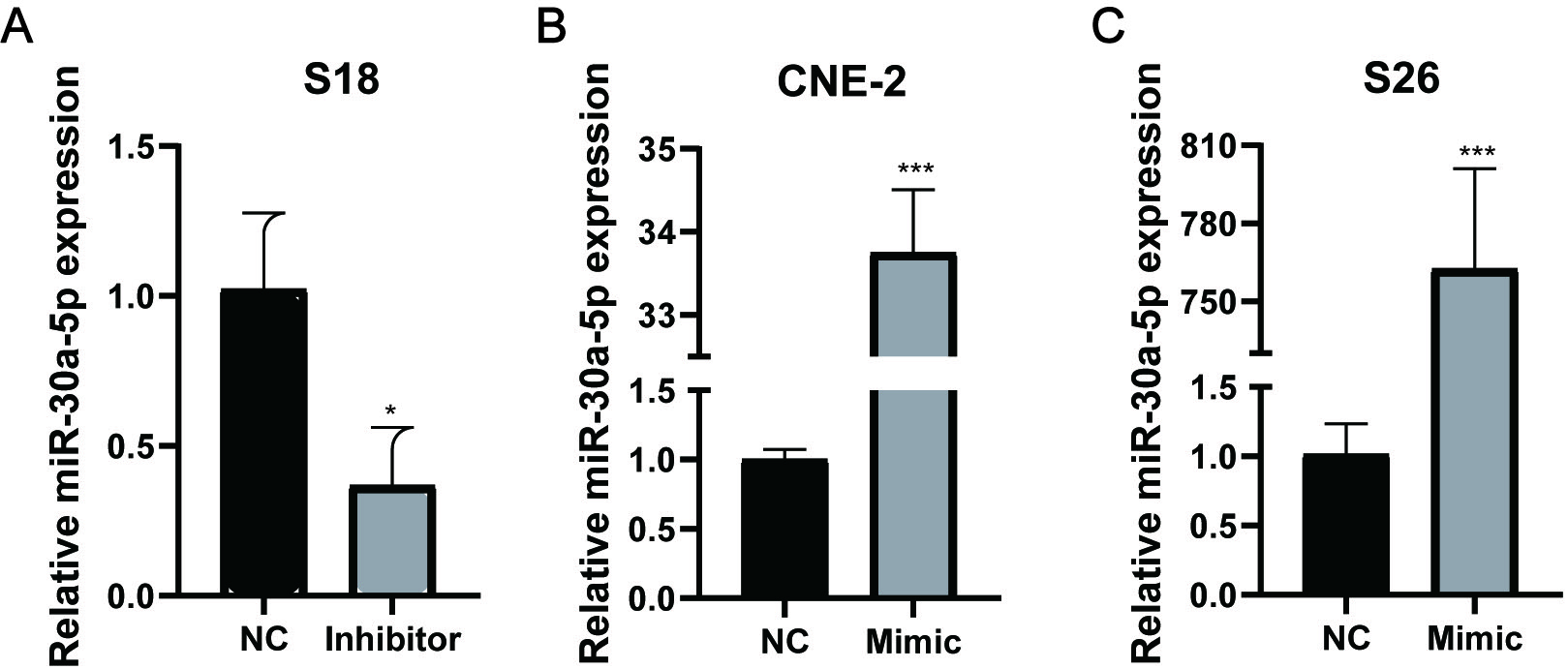


**Supplementary Figure 1. Relative expression of miR-30a-5p after genetic interventions.** Relative expression of miR-30a-5p in S18 cells transfected with miR-30a-5p inhibitor (A), and in CNE-2 (B) and S26 (C) cells transfected with miR-30a-5p mimic.
