# Supplementary material for: Communication between cancer cell subtypes by exosomes contributes to nasopharyngeal carcinoma metastasis and poor prognosis
Source: Precis Clin Med. 2024 Sep 23;7(3):pbae018. doi: 10.1093/pcmedi/pbae018 (PMC11427951; doi:10.1093/pcmedi/pbae018)
Supplement: pbae018_Supplemental_Files [file pbae018_supplemental_files.zip › supplementary_table_1.docx]

**Supplementary Table 1.** Significantly enriched miRNA in exosomes derived from S18 cells compared with exosomes from CNE-2 and S26 cell.

| MiRNA | S18  (RPM) | Vs. CNE-2 | | |  | Vs. S26 | | |
| --- | --- | --- | --- | --- | --- | --- | --- | --- |
|  |  | RPM | log2 (fold change) | *P*-value |  | RPM | log2 (fold change) | *P*-value |
| hsa-miR-30a-5p | 24870.79 | 6394.72 | 1.9595 | 9.96E-08 |  | 7945.013 | 1.6463 | 8.24E-06 |
| hsa-miR-320a | 12105.76 | 25957.35 | -1.1004 | 0.003254 |  | 34675.48 | -1.5182 | 1.11E-05 |
| hsa-miR-148b-3p | 6372.747 | 1869.65 | 1.7691 | 2.73E-07 |  | 679.27 | 3.2299 | 1.39E-20 |
| hsa-miR-22-3p | 4671.897 | 1733.977 | 1.4299 | 6.35E-06 |  | 1443.977 | 1.694 | 1.86E-07 |
| hsa-miR-122-5p | 3878.233 | 15255.49 | -1.9759 | 1.67E-07 |  | 21690.73 | -2.4836 | 3.12E-13 |
| hsa-miR-320c | 1689.373 | 831.1767 | 1.0233 | 0.004974 |  | 771.7667 | 1.1303 | 0.002228 |
| hsa-miR-582-3p | 1364.063 | 540.0633 | 1.3367 | 0.000132 |  | 275.37 | 2.3085 | 4.99E-15 |
| hsa-miR-320d | 1347.857 | 428.1133 | 1.6546 | 3.30E-05 |  | 309.7033 | 2.1217 | 8.81E-07 |
| hsa-miR-146b-5p | 1118.617 | 530.6267 | 1.0759 | 4.83E-05 |  | 312.9333 | 1.8378 | 4.19E-10 |
| hsa-miR-181b-5p | 1098.947 | 360.2767 | 1.6089 | 9.69E-07 |  | 496.56 | 1.1461 | 0.000235 |
| hsa-miR-149-5p | 953.0267 | 440.0467 | 1.1149 | 0.000161 |  | 444.69 | 1.0997 | 0.00023 |
| hsa-miR-378c | 851.5867 | 392.2533 | 1.1184 | 0.001779 |  | 269.9033 | 1.6577 | 2.83E-05 |
| hsa-miR-221-5p | 701.3967 | 248.9967 | 1.4941 | 3.87E-05 |  | 293.74 | 1.2557 | 4.45E-05 |
| hsa-miR-451a | 576.0467 | 1907.91 | -1.7277 | 4.91E-05 |  | 2252.25 | -1.9671 | 3.32E-06 |
| hsa-miR-574-5p | 548.37 | 182.9167 | 1.584 | 0.00012 |  | 72.9033 | 2.9111 | 7.19E-09 |
| hsa-miR-10b-5p | 487.8867 | 1091.147 | -1.1612 | 0.001769 |  | 2748.687 | -2.4941 | 1.60E-08 |
| hsa-miR-378d | 410.27 | 96.6867 | 2.0852 | 2.50E-07 |  | 139.25 | 1.5589 | 3.67E-05 |
| hsa-miR-503-5p | 383.4633 | 83.4433 | 2.2002 | 3.49E-09 |  | 38.44 | 3.3184 | 1.67E-15 |
| hsa-miR-200c-3p | 230.8667 | 22062.6 | -6.5784 | 1.29E-68 |  | 24107.69 | -6.7063 | 5.33E-56 |
| hsa-miR-127-3p | 204.1467 | 529.41 | -1.3748 | 0.000425 |  | 2298.937 | -3.4933 | 4.31E-18 |
| hsa-miR-381-3p | 137.45 | 403.09 | -1.5522 | 3.51E-06 |  | 498.0467 | -1.8574 | 6.11E-07 |
| hsa-miR-7704 | 105.9167 | 568.3133 | -2.4238 | 0.000113 |  | 2181.777 | -4.3645 | 4.87E-19 |
| hsa-miR-7641 | 97.37 | 771.5367 | -2.9862 | 1.97E-08 |  | 748.35 | -2.9422 | 2.59E-09 |
| hsa-miR-495-3p | 66.6933 | 212.0633 | -1.6689 | 3.00E-05 |  | 239.48 | -1.8443 | 3.73E-05 |
| hsa-miR-200b-3p | 21.13 | 860.0533 | -5.3471 | 1.18E-33 |  | 611.1067 | -4.8541 | 2.19E-25 |
| hsa-miR-205-5p | 1.0967 | 7555.117 | -12.7501 | 9.37E-83 |  | 3639.867 | -11.6965 | 4.94E-61 |

RPM: Reads Per Million
