# Supplementary material for: Communication between cancer cell subtypes by exosomes contributes to nasopharyngeal carcinoma metastasis and poor prognosis
Source: Precis Clin Med. 2024 Sep 23;7(3):pbae018. doi: 10.1093/pcmedi/pbae018 (PMC11427951; doi:10.1093/pcmedi/pbae018)
Supplement: pbae018_Supplemental_Files [file pbae018_supplemental_files.zip › supplementary_table_2.docx]

**Supplementary Table 2.** Clinical information of the selected NPC patients

|  | miR-30a-5p level^*^ | |  |
| --- | --- | --- | --- |
| Characteristic | Low,  No. of patients (%) | High,  No. of patients (%) | *P*-value |
| Total | 60 | 59 |  |
| Age,y |  |  | 0.647 |
| Median | 47.5 | 50 |  |
| Range | (21-78) | (28-76) |  |
| Sex |  |  | 0.225 |
| Female | 18(30.0%) | 12(20.3%） |  |
| Male | 42(70.0%） | 47(79.7%) |  |
| EBV DNA |  |  | 0.163 |
| <4000copies/mL | 41(68.3%) | 33(55.9%) |  |
| ≥4000copies/mL | 19(31.7%) | 26(44.1%) |  |
| T stage |  |  | 0.559 |
| T1 | 5(8.3%) | 4(6.8%) |  |
| T2 | 9(15.0%) | 7(11.9%) |  |
| T3 | 30(50.0%) | 25(42.4%) |  |
| T4 | 16(26.7%) | 23(39.0%) |  |
| N stage |  |  | 0.688 |
| N0 | 16(26.7%) | 13(22.0%) |  |
| N1 | 16(26.7%) | 19(32.2%) |  |
| N2 | 20(33.3%) | 16(27.1%) |  |
| N3 | 8(13.3%) | 11(18.6%) |  |
| Overall stage |  |  | 0.203 |
| I | 2(3.3%) | 2(3.4%) |  |
| II | 7(11.7%) | 5(8.5%) |  |
| III | 30(50.0%) | 20(33.9%) |  |
| IVa | 21(25.0%) | 32(54.2%) |  |

* Patients were divided into two groups by the median value of plasma exosomal miR-30a-5p expression level.
