# Supplementary material for: Communication between cancer cell subtypes by exosomes contributes to nasopharyngeal carcinoma metastasis and poor prognosis
Source: Precis Clin Med. 2024 Sep 23;7(3):pbae018. doi: 10.1093/pcmedi/pbae018 (PMC11427951; doi:10.1093/pcmedi/pbae018)
Supplement: pbae018_Supplemental_Files [file pbae018_supplemental_files.zip › supplementary_table_3.docx]

**Supplementary Table 3.** Multivariate analyses of risk factors in the selected NPC patients

|  |  | OS | |  | PFS | |  | DMFS | |  | LRFS | |
| --- | --- | --- | --- | --- | --- | --- | --- | --- | --- | --- | --- | --- |
| Characteristic | No. of patients (%) | HR (95% CI) | *P*-value |  | HR (95% CI) | *P*-value |  | HR (95% CI) | *P*-value |  | HR (95% CI) | *P*-value |
| Age |  |  |  |  |  |  |  |  |  |  |  |  |
| <60 | 92(77.3%) | Reference |  |  | Reference |  |  | Reference |  |  | Reference |  |
| ≥60 | 27(22.7%) | 0.785 (0.266-2.312) | 0.785 |  | 1.205 (0.592-2.457) | 0.607 |  | 1.656 (0.703-3.899) | 0.249 |  | 0.766 (0.233-2.514) | 0.660 |
| Sex |  |  |  |  |  |  |  |  |  |  |  |  |
| Female | 30(25.2%) | Reference |  |  | Reference |  |  | Reference |  |  | Reference |  |
| Male | 89(74.8%) | 0.858 (0.263-2.797) | 0.799 |  | 0.868 (0.413-1.824) | 0.708 |  | 1.050 (0.410-2.688) | 0.919 |  | 1.090 (0.288-4.131) | 0.899 |
| EBV DNA |  |  |  |  |  |  |  |  |  |  |  |  |
| <4000copies/mL | 74(62.2%) | Reference |  |  | Reference |  |  | Reference |  |  | Reference |  |
| ≥4000copies/mL | 45(37.8%) | 1.324 (0.519-3.376) | 0.557 |  | 1.115 (0.580-2.145) | 0.744 |  | 0.646 (0.277-1.505) | 0.311 |  | 1.432 (0.516-3.970) | 0.490 |
| T stage |  |  |  |  |  |  |  |  |  |  |  |  |
| T1-2 | 25(21.0%) | Reference |  |  | Reference |  |  | Reference |  |  | Reference |  |
| T3 | 55(46.2%) | 1.534 (0.321-7.331) | 0.592 |  | 1.030 (0.414-2.561) | 0.949 |  | 0.978 (0.327-2.923) | 0.969 |  | 0.629 (1.150-2.640) | 0.526 |
| T4 | 39(32.8%) | 4.629 (0.988-21.688) | 0.052 |  | 2.356 (0.965-5.748) | 0.060 |  | 1.986 (0.686-5.744) | 0.206 |  | 1.449 (0.359-5.853) | 0.603 |
| N stage |  |  |  |  |  |  |  |  |  |  |  |  |
| N0 | 29(24.4%) | Reference |  |  | Reference |  |  | Reference |  |  | Reference |  |
| N1 | 35(29.4%) | 3.264 (0.356-29.930) | 0.295 |  | 2.944 (0.938-9.244) | 0.064 |  | 3.394 (0.699-16.484) | 0.130 |  | 3.053 (0.604-15.427) | 0.177 |
| N2 | 36(30.3%) | 11.561 (1.456-91.797) | 0.021 |  | 5.181 (1.737-15.448) | 0.003 |  | 7.626 (1.691-34.399) | 0.008 |  | 3.336 (0.652-17.068) | 0.148 |
| N3 | 19(16.0%) | 18.095 (2.125-154.061) | 0.008 |  | 7.011 (2.108-23.323) | 0.001 |  | 16.344 (3.303-80.871) | 0.001 |  | 1.889 (0.245-14.584) | 0.542 |
| miR-30a-5p level* |  |  |  |  |  |  |  |  |  |  |  |  |
| Low | 60(50.4%) | Reference |  |  | Reference |  |  | Reference |  |  | Reference |  |
| High | 59(49.6%) | 2.333 (0.928-5.865) | 0.072 |  | 1.976 (1.061-3.680) | 0.032 |  | 2.245 (1.034-4.872) | 0.041 |  | 2.569 (0.874-7.555) | 0.086 |

* Patients were divided into two groups by the median value of plasma exosomal miR-30a-5p expression level.

Abbreviations: OS, overall survival; PFS, progression-free survival; DMFS, distant metastasis-free survival; LRFS, locoregional recurrence-free survival; CI, confidence interval; HR, hazard ratio.
